# Intermittent pneumatic compression *versus* additional prophylaxis with enoxaparin for prevention of venous thromboembolism after laparoscopic surgery for gastric and colorectal malignancies: multicentre randomized clinical trial

**DOI:** 10.1002/bjs5.50323

**Published:** 2020-07-23

**Authors:** H. Kamachi, S. Homma, H. Kawamura, T. Yoshida, Y. Ohno, N. Ichikawa, R. Yokota, T. Funakoshi, Y. Maeda, N. Takahashi, T. Amano, A. Taketomi

**Affiliations:** ^1^ Department of Gastroenterological Surgery I, Graduate School of Medicine Hokkaido University Hokkaido Japan; ^2^ Department of Gastrointestinal Surgery, National Hospital Organization Hokkaido Cancer Centre Hokkaido; ^3^ Surgical Centre Hokkaido Japan; ^4^ Clinical Research and Medical Innovation Centre Hokkaido University Hospital Hokkaido Japan; ^5^ Department of Surgery Sunagawa City Medical Centre Sunagawa Japan; ^6^ Department of Surgery Asahikawa‐Kosei General Hospital Asahikawa Japan

## Abstract

**Background:**

The role of antithrombotic chemoprophylaxis in prevention of venous thromboembolism (VTE) in laparoscopic surgery for gastric and colorectal malignancies is unknown. This study compared the addition of enoxaparin following intermittent pneumatic compression (IPC) with IPC alone in patients undergoing laparoscopic surgery for gastrointestinal malignancy.

**Methods:**

In this multicentre RCT, eligible patients were older than 40 years and had a WHO performance status of 0 or 1. Exclusion criteria were prescription of antiplatelet or anticoagulant drugs and history of VTE. Patients were allocated to IPC or to ICP with enoxaparin in a 1 : 1 ratio. Stratification factors included sex, location of cancer, age 61 years and over, and institution. Enoxaparin was administered on days 1–7 after surgery. Primary outcome was VTE, evaluated by multidetector CT on day 7.

**Results:**

Of 448 patients randomized, 208 in the IPC group and 182 in the IPC with enoxaparin group were evaluated. VTE occurred in ten patients (4·8 per cent) in the IPC group and six (3·3 per cent) in the IPC with enoxaparin group (*P* = 0·453). Proximal deep vein thrombosis and/or pulmonary embolism occurred in seven patients (3·4 per cent) in the IPC group and one patient (0·5 per cent) in the IPC with enoxaparin group (*P* = 0·050). All VTE events were asymptomatic and non‐fatal. Bleeding occurred in 11 of 202 patients in the IPC with enoxaparin group, and one patient needed a transfusion. All bleeding events were managed by discontinuation of the drug.

**Conclusion:**

IPC with enoxaparin after laparoscopic surgery for gastric and colorectal malignancies did not reduce the rate of VTE. Registration number: UMIN000011667 ( https://www.umin.ac.jp/).

## Graphical abstract

This RCT compared intermittent pneumatic compression (IPC) alone with IPC plus enoxaparin for 1 week after surgery in the prevention of venous thromboembolism (VTE) in patients undergoing laparoscopic surgery for gastric or colorectal malignancy. Although VTE occurred in 4·8 per cent of the ICP‐alone group and 3·3 per cent of the ICP with enoxaparin group (*P* = 0·453), the occurrence of proximal deep vein thrombosis and/or pulmonary embolism was reduced from 3·4 to 0·5 per cent by the addition of enoxaparin (*P* = 0·050).

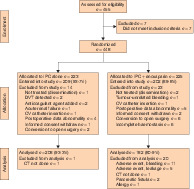

No additional value of enoxaparin in patients who recover uneventful

## Abstract (es)

**Antecedentes:**

El papel de la quimioprofilaxis para la prevención del tromboembolismo venoso (*venous thromboembolism*, VTE) en la cirugía laparoscópica de los tumores malignos gástricos y colorrectales se desconoce. El objetivo de este estudio fue comparar la quimioprofilaxis antitrombótica (enoxaparina) y la compresión neumática intermitente (*intermittent pneumatic compression*, IPC) en pacientes sometidos a cirugía laparoscópica de tumores malignos abdominales.

**Métodos:**

Se efectuó un ensayo aleatorizado, controlado y multicéntrico de pacientes sometidos a cirugía laparoscópica de tumores gástricos y colorrectales en Japón. Los criterios de inclusión eran pacientes mayores de 40 años de edad y con un estado funcional de WHO de 0‐1. Los criterios de exclusión fueron la prescripción al paciente de fármacos antiagregantes o anticoagulantes y la historia de VTE. Los pacientes fueron asignados a IPC y ICP con la adición de enoxaparina en una relación 1:1. Los factores de estratificación incluyeron el sexo, la localización del cáncer, la edad mayor o menor de 61 años, y la institución. La enoxaparina fue administrada en los días postoperatorios (*postoperative day*, POD) 1‐7. El resultado primario fue la VTE evaluada mediante tomografía computarizada multidetector en el POD7. Los cálculos de la potencia determinaron que se requerían 184 pacientes en cada grupo.

**Resultados:**

De los 448 pacientes aleatorizados, se evaluaron finalmente 208 pacientes en el grupo IPC y 182 pacientes en el grupo IPC más enoxaparina. La VTE ocurrió en 10 de 208 pacientes en el grupo IPC (4,8%) y 6 de 182 pacientes en el grupo IPC más enoxaparina (3,3%) (*P* = 0,45). La trombosis venosa profunda proximal (*proximal deep vein thrombosis*, DVT) y/o el embolismo pulmonar (*pulmonary embolism*, PE) ocurrieron en 7 de 208 pacientes en el grupo IPC (3,4%) y 1 de 182 pacientes en el grupo IPC más enoxaparina (0,55%) (riesgo relativo 0,163, i.c. del 95% 0,020‐1,314, *P* = 0,0503). Todos los eventos de VTE fueron asintomáticos y no mortales. Se produjo una hemorragia en 11 de 202 pacientes en el grupo IPC con enoxaparina (5,4%, i.c. del 95% 3,1%‐9,5%, *P* < 0,001), y un paciente precisó transfusión. Todos los eventos hemorrágicos pudieron ser tratados con la interrupción del fármaco.

**Conclusión:**

La IPC con la adición de enoxaparina tras cirugía laparoscópica de los tumores malignos gástricos y colorrectales no disminuye la VTE.

## Introduction

Venous thromboembolism (VTE), including deep vein thrombosis (DVT) and pulmonary embolism (PE), is one of the most critical postoperative complications. Between 15 and 40 per cent of surgical patients develop VTE, and 0·8 per cent of those patients will develop fatal PE without receiving antithrombotic prophylaxis^1^. Meta‐analyses^2^, ^3^, ^4^, ^5^ of RCTs have shown that antithrombotic prophylaxis reduces the incidence of VTE.

According to the VTE risk score after surgery^6^, ^7^, the guidelines of the American College of Chest Physicians (ACCP)^8^ and the American Society of Clinical Oncology^9^ recommend the use of antithrombotic chemoprophylaxis after major abdominal cancer surgery. For laparoscopic surgery, the Society of American Gastrointestinal and Endoscopic Surgeons (SAGES)^10^ recommends following the ACCP guidelines. SAGES also mentions the need for further studies to establish antithrombotic chemoprophylaxis for VTE after laparoscopic surgery, because there was a statistically significant reduction in the risk of VTE after laparoscopic compared with open surgery^11^.

The aim of this study was to assess the additional effect of antithrombotic chemoprophylaxis with enoxaparin to prevent VTE in patients undergoing laparoscopic surgery for gastrointestinal cancer.

## Methods

In this multicentre RCT, patients with gastric or colorectal cancer scheduled for laparoscopic surgery in 15 regional Japanese hospitals in Hokkaido prefecture between October 2013 and October 2017 were eligible for inclusion. All hospitals were certified facilities of the Japan Surgical Society and the Japanese Society of Gastroenterological Surgery, and all surgeons were board‐certified in gastroenterology and/or qualified surgeons of the Japan Society for Endoscopic Surgery. Inclusion criteria were patients older than 40 years with WHO performance status 0 or 1, who agreed to participate in the study. Exclusion criteria were a history of heparin‐induced thrombocytopenia or heparin hypersensitivity, acute bacterial endocarditis, creatinine clearance below 50 ml/min, severe hepatic dysfunction (Child grade C), weight less than 40 kg, pregnancy, prescription of antiplatelet or anticoagulant drugs, history of venous thromboembolic disease within 1 year, history of hypersensitivity for iodinated contrast agent, presence of central venous catheter, treatment with oestrogen or progesterone within 4 weeks of the operation, and radiotherapy or chemotherapy within 2 weeks of surgery. Preoperative examination by ultrasonography or CT to rule out the presence of DVT/PE was performed in patients with a preoperative d‐dimer value of 1·0 μg/ml or above. Patients who met these inclusion and exclusion criteria were randomized after registration.

Patients whose condition changed while waiting for surgery and who then met the exclusion criteria were excluded. Patients who met any of the following criteria after surgery were excluded from evaluation: patients not treated, those with peritoneal dissemination, conversion to open surgery, haemoglobin level below 9·5 g/dl, alanine or aspartate aminotransferase values more than 2·5 times the normal values for the facility, total bilirubin concentration above 3·0 mg/dl, oxygen saturation less than 90 per cent, incomplete haemostasis (defined by persistent bleeding from abdominal drain or gastrointestinal tract), and withdrawal of consent. In addition, patients who were withdrawn from the study because of adverse events and complications were excluded. The final VTE evaluation was carried out for patients who completed the study protocol. Patients excluded from the study after surgery were treated without enoxaparin. The study was conducted in an open‐label fashion.

### Ethical approval

The study was performed with the approval of the Internal Review Board on ethical issues of Hokkaido University Hospital and participating sites. All procedures performed in studies involving human participants were in accordance with the ethical standards of the institutional and/or national research committee, and with the 1964 Declaration of Helsinki and its later amendments or comparable ethical standards. Written informed consent was obtained from all individual participants included in the study. The study was registered at the UMIN Clinical Trials Registry System (https://www.umin.ac.jp/ctr/; ID UMIN000011667).

### Randomization

Physicians designated by each hospital registered the patients, and central computer‐generated randomization was performed by the research coordinators of Hokkaido University Hospital's Clinical Research and Medical Innovation Centre. Patients were allocated to intermittent pneumatic compression (IPC) or to IPC with enoxaparin in a 1 : 1 ratio at least 1 day before surgery. Stratification factors included sex, location of cancer (stomach, colon or rectum), age 61 years or more, and institution.

Blinding was considered to be impossible in this study.

**Fig. 1:**
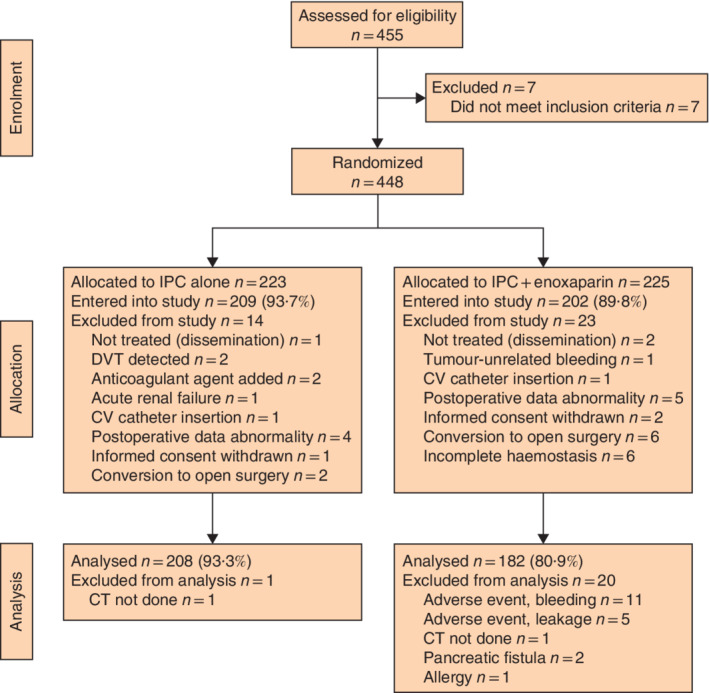
CONSORT diagram for the study IPC, intermittent pneumatic compression; DVT, deep vein thrombosis; CV, central venous.

### Study procedures

All operations were performed by standard multiport laparoscopic surgery with pneumoperitoneum pressure from 12 to 8 mmHg. The lithotomy position was done for the colorectal operation, and linear and circular staplers were used for gastrointestinal anastomoses. The postoperative recovery protocol was according to enhanced recovery after surgery (ERAS) principles. The IPC system was applied to both legs after induction of anaesthesia and before patient positioning. Calf‐length sleeves were used. IPC was continued until leaving the bed on the first postoperative day (POD1). Enoxaparin was administered from 24 h after surgery (subcutaneous injection of 2000 units twice daily on POD1–7). In patients in the IPC with enoxaparin group who also had epidural anaesthesia, administration of enoxaparin was discontinued for 12 h before and after removal of the epidural tube.

### Outcome

The primary outcome was VTE (DVT and PE), both symptomatic and asymptomatic, diagnosed by multidetector CT on POD7. DVT located below the knee and confined to the calf veins was defined as distal DVT, and that located in the popliteal, femoral or iliac veins was defined as proximal DVT. The radiologist in each hospital evaluated the occurrence of VTE with no information on patient allocation.

The secondary outcome was bleeding that occurred by POD7. Postoperative bleeding was classified as major or minor, defined according to the International Society on Thrombosis and Haemostasis criteria^12^. Major bleeding was defined as intracranial, retroperitoneal or clinically overt haemorrhage associated with a decrease in the haemoglobin level of more than 20 g/l, transfusion of two or more units of packed cells, or need for surgical intervention. Minor bleeding was defined as clinically relevant non‐major bleeding associated with medical intervention. All bleeding events were reviewed by the Internal Review Board at each participating site. Patients with adverse events and complications by POD7 that led to study discontinuation were excluded from the final evaluation.

### Data collection

Study data, including patient characteristics, medical history, laboratory data, cancer type and staging, surgical information and outcome, and VTE events, were transferred to the central data collection centre.

### Sample size calculation

The incidence of VTE for high‐risk gastrointestinal surgery is 20–25 per cent^1^, and laparoscopic surgery reduces the incidence by about one‐half^11^. The estimated risk of VTE was therefore 10·0 per cent in the IPC group. The estimated risk of VTE in the IPC plus enoxaparin group was 2 per cent^13^, implying an 8 per cent reduction. With an α of 0·05 and β of 0·10, 368 patients had to be evaluable in the study. The anticipated dropout rate due to adverse events in either group was 20 per cent. Therefore, 450 patients had to be recruited for the study.

### Statistical analysis

Statistical analysis was performed using JMP® Pro 13.0.0 for Windows® (SAS Institute, Cary, North Carolina, USA). Categorical data were compared with the χ^2^ test, and continuous or ordinal data using the Mann–Whitney *U* test. *P* < 0·050 (two‐sided) was considered to denote statistical significance. The analysis was not done according to the intention‐to‐treat principle.

## Results

Of 455 patients who were offered participation in the study, 448 were registered and randomized. After postrandomization exclusion, 209 patients remained in the IPC group and 202 in the IPC with enoxaparin group (*Fig*. *1*). Following exclusion for postoperative complications, 208 and 182 patients respectively in these two groups remained in the study for evaluation of the primary endpoint. Patient demographics and clinical characteristics of evaluable patients in the two groups were comparable (*Table* *1*). The surgical characteristics (surgical procedure, cancer type, cancer stage according to the TNM classification, duration of surgery, and use of the lithotomy position during surgery) of evaluable patients showed no statistical differences between the two groups.

**Table 1:** Patient demographics and clinical details of randomized and treated patients

	IPC alone	IPC with enoxaparin	*P* §
**Randomized patients**	*n* = 223	*n* = 225	
Age (years)*	65·1(9·3)	65·0(9·2)	
Age ≥ 61 years	164 (73·5)	164 (72·9)	
Male sex	135 (60·5)	135 (60·0)	
Location of cancer			
Stomach	74 (33·2)	80 (35·6)	
Colon	88 (39·5)	94 (41·8)	
Rectum	61 (27·4)	51 (22·7)	
**Evaluable patients**	*n* = 208	*n* = 182	
Age (years)*	65·0(9·3)	64·8(9·1)	0·670¶
Age ≥ 61 years	153 (73·6)	132 (72·5)	0·819
Male sex	125 (60·1)	106 (58·2)	0·710
ASA grade			
I	108 (51·9)	94 (51·6)	0·957
II	99 (47·6)	86 (47·3)	0·946
III	1 (0·5)	2 (1·1)	0·486
BMI (kg/m^2^)*	23·3(3·3)	23·7(3·7)	0·473¶
BMI > 30 kg/m^2^	6 (2·9)	10 (5·5)	0·113
Location of cancer			
Stomach	66 (31·7)	65 (35·7)	0·406
Colon	85 (40·9)	77 (42·3)	0·773
Rectum	57 (27·4)	40 (22·0)	0·216
Gastric surgery	66 (31·7)	65 (35·7)	0·406
Total gastrectomy	15 (7·2)	12 (6·6)	0·810
Distal gastrectomy	51 (24·5)	50 (27·5)	0·507
Partial gastrectomy‡	0 (0)	3 (1·6)	0·063
Colorectal surgery	142 (68·3)	117 (64·3)	0·406
Right hemicolectomy	30 (14·4)	27 (14·8)	0·909
Left hemicolectomy	9 (4·3)	4 (2·2)	0·243
Sigmoid resection	27 (13·0)	33 (18·1)	0·160
Subtotal colectomy	1 (0·5)	0 (0)	0·349
Other colectomy	16 (7·7)	9 (4·9)	0·269
Anterior resection	51 (24·5)	37 (20·3)	0·323
Abdominoperineal resection	8 (3·8)	5 (2·7)	0·546
Other rectal resection	0 (0)	2 (1·1)	0·130
Cancer stage			
0	2 (1·0)	2 (1·1)	0·893
1	86 (41·3)	82 (45·1)	0·461
2	62 (29·8)	52 (28·6)	0·789
3	51 (24·5)	33 (18·1)	0·126
4	4 (1·9)	12 (6·6)	0·020
Undefined	3 (1·4)	1 (0·5)	0·383
Duration of surgery (min)†	220 (82–484)	230·5 (65–435)	0·566¶
Lithotomy position	113 (54·3)	100 (54·9)	0·903

Values in parentheses are percentages unless indicated otherwise; values are

* mean(s.d.) and

† median (range).

‡ Includes wedge resection and remnant gastrectomy. IPC, intermittent pneumatic compression.

§ χ^2^ test, except

¶ Mann–Whitney *U* test.

### Thromboembolic events

All VTE events were asymptomatic and not fatal. Ten of the 208 patients in the IPC group had a VTE event (4·8 (95 per cent c.i. 2·6 to 8·6) per cent), compared with six of the 182 patients in the IPC with enoxaparin group (3·3 (1·5 to 7·0) per cent) (*P* = 0·453) (*Table* *2*).

**Table 2:** Venous thromboembolism in evaluable patients

	Total (*n* = 390)	IPC alone (*n* = 208)	IPC with enoxaparin (*n* = 182)	P*
Overall VTE	16 (4·1)	10 (4·8)	6 (3·3)	0·453
Distal DVT	10 (2·6)	5 (2·4)	5 (2·7)	0·831
Proximal DVT	1 (0·3)	1 (0·5)	0 (0)	0·349
PE	7 (1·8)	6 (2·9)	1 (0·5)	0·083
Proximal DVT or PE	8 (2·1)	7 (3·4)	1 (0·5)	0·050

Values in parentheses are percentages. IPC, intermittent pneumatic compression; VTE, venous thromboembolism; DVT, deep vein thrombosis; PE, pulmonary embolism. *χ^2^ test.

### Bleeding complications and adverse events

All 208 patients in the IPC group had an uneventful course and completed the study. Bleeding complications were observed in 11 of 202 patients (5·4 (95 per cent c.i. 3·1 to 9·5) per cent) in the IPC with enoxaparin group. Ten patients had minor bleeding and one patient had major bleeding requiring a blood transfusion. Bleeding sites included blood loss via the abdominal drain (3 patients), subcutaneous bleeding (4) and anastomosis bleeding (4); bleeding events occurred a mean(s.d.) of 3·5(1·4) days after surgery. Haemostasis was obtained in all patients with drug discontinuation, and none required an additional intervention. Bleeding did not lead to death.

During follow‐up of up to 30 days after surgery, no signs of symptomatic VTE were seen. In addition to the five patients (2·5 per cent) with anastomotic leakage in the IPC with enoxaparin group (*Fig*. *1*), surgical‐site infection (SSI) occurred in five (1·3 per cent) of 385 evaluable patients (3 superficial and 2 organ/space SSI).

## Discussion

Compared with IPC alone, IPC with postoperative administration of enoxaparin for 1 week did not significantly reduce VTE after laparoscopic surgery for gastric or colorectal malignancy.

The incidence of VTE after laparoscopic surgery is lower than that after open surgery^11^, but methods for diagnosis of VTE vary from examination of only symptomatic cases to examination with active screening of lower‐limb ultrasonography and/or venography. In the literature, the incidence of VTE after laparoscopic gastric surgery, including bariatric surgery, ranges from 0 to 19·4 per cent^14^, ^15^, ^16^, ^17^, ^18^, and that after laparoscopic colorectal surgery ranges from 0·8 to 17·1 per cent^18^, ^19^, ^20^, ^21^, ^22^, ^23^, ^24^, ^25^. By active screening with duplex ultrasonography and either ventilation–perfusion scanning or CT angiography in injured patients, the rate of DVT increases tenfold and the rate of PE almost fivefold^26^. Multidetector CT has high diagnostic accuracy for PE and DVT, with a sensitivity of 90 per cent and specificity of 95 per cent for PE, and values of 97 and 100 per cent respectively for DVT^27^, ^28^.

In the present study, the observed incidence of diagnosis of perioperative VTE by multidetector CT in patients whose operation was completed laparoscopically and who had no serious adverse events during the first week was lower than expected. Sakon and colleagues^13^ reported that the incidence of VTE diagnosed by active screening of lower‐limb venography after major abdominal surgery was 19·4 per cent with no antithrombotic chemoprophylaxis. Symptomatic VTE had not occurred. Laparoscopic *versus* open surgery and mobilization of patients according to an ERAS protocol may have influenced the incidence of VTE in the IPC‐alone group.

Additional morbidity associated with enoxaparin was limited. This study was not powered to detect a difference in proximal venous thrombosis and PE. In patients with additional risk factors for VTE, including obesity and advanced cancer, chemoprophylaxis should be considered, despite the negative findings in this study.

This study has some limitations. The primary endpoint was not evaluated in all patients who were randomized owing to conversion to open surgery and complications. Nothing can be concluded regarding the efficacy of thrombotic prophylaxis in these patients, and their exclusion may have influenced the results. The overestimated incidence of VTE with no thrombotic prophylaxis in laparoscopic surgery for colorectal and gastric cancer in the study protocol led to a study that was underpowered to assess a statistically significant difference between groups. Caution should be exercised in generalizing the results of this study to the rest of the world, as all patients were Asian, whose population‐wide VTE incidence is approximately 15–20 per cent of the levels recorded in Western countries^29^. In addition, the mean BMI of patients was low, and early‐stage cancer was common.

## References

[1] Geerts WH , Heit JA , Clagett GP , Pineo GF , Colwell CW , Anderson FA Jr *et al* Prevention of venous thromboembolism. Chest 2001; 119: 132S–175S. 11157647 10.1378/chest.119.1_suppl.132s

[2] Clagett GP , Reisch JS . Prevention of venous thromboembolism in general surgical patients. Results of meta‐analysis. Ann Surg 1988; 208: 227–240. 2456748 10.1097/00000658-198808000-00016 PMC1493611

[3] Collins R , Scrimgeour A , Yusuf S , Peto R. Reduction in fatal pulmonary embolism and venous thrombosis by perioperative administration of subcutaneous heparin. Overview of results of randomized trials in general, orthopedic, and urologic surgery. N Engl J Med 1988; 318: 1162–1173. 3283548 10.1056/NEJM198805053181805

[4] Mismetti P , Laporte S , Darmon JY , Buchmuller A , Decousus H. Meta‐analysis of low molecular weight heparin in the prevention of venous thromboembolism in general surgery. Br J Surg 2001; 88: 913–930. 11442521 10.1046/j.0007-1323.2001.01800.x

[5] Zareba P , Wu C , Agzarian J , Rodriguez D , Kearon C. Meta‐analysis of randomized trials comparing combined compression and anticoagulation with either modality alone for prevention of venous thromboembolism after surgery. Br J Surg 2014; 101: 1053–1062. 24916118 10.1002/bjs.9527

[6] Rogers SO Jr , Kilaru RK , Hosokawa P , Henderson WG , Zinner MJ , Khuri SF . Multivariable predictors of postoperative venous thromboembolic events after general and vascular surgery: results from the Patient Safety in Surgery Study. J Am Coll Surg 2007; 204: 1211–1221. 17544079 10.1016/j.jamcollsurg.2007.02.072

[7] Caprini JA . Risk assessment as a guide for the prevention of the many faces of venous thromboembolism. Am J Surg 2010; 199: S3–S10. 20103082 10.1016/j.amjsurg.2009.10.006

[8] Gould MK , Garcia DA , Wren SM , Karanicolas PJ , Arcelus JI , Heit JA *et al* Prevention of VTE in nonorthopedic surgical patients: antithrombotic therapy and prevention of thrombosis, 9th ed: American College of Chest Physicians Evidence‐Based Clinical Practice Guidelines. Chest 2012; 141: e227S–e277S. 22315263 10.1378/chest.11-2297 PMC3278061

[9] Lyman GH , Bohlke K , Khorana AA , Kuderer NM , Lee AY , Arcelus JI *et al* Venous thromboembolism prophylaxis and treatment in patients with cancer: American Society of Clinical Oncology Clinical Practice Guideline update 2014. J Clin Oncol 2015; 33: 654–656. 25605844 10.1200/JCO.2014.59.7351 PMC4881372

[10] Richardson WS , Hamad GG , Stefanidis D . SAGES VTE prophylaxis for laparoscopic surgery guidelines: an update. Surg Endosc 2017; 31: 501–503. 28091748 10.1007/s00464-016-5402-z

[11] Nguyen NT , Hinojosa MW , Fayad C , Varela E , Konyalian V , Stamos MJ *et al* Laparoscopic surgery is associated with a lower incidence of venous thromboembolism compared with open surgery. Ann Surg 2007; 246: 1021–1027. 18043105 10.1097/SLA.0b013e31815792d8

[12] Schulman S , Angeras U , Bergqvist D , Eriksson B , Lassen MR , Fisher W ; Subcommittee on Control of Anticoagulation of the Scientific and Standardization Committee of the International Society on Thrombosis and Haemostasis. Definition of major bleeding in clinical investigations of antihemostatic medicinal products in surgical patients. J Thromb Haemost 2010; 8: 202–204. 19878532 10.1111/j.1538-7836.2009.03678.x

[13] Sakon M , Kobayashi T , Shimazui T . Efficacy and safety of enoxaparin in Japanese patients undergoing curative abdominal or pelvic cancer surgery: results from a multicenter, randomized, open‐label study. Thromb Res 2010; 125: e65–e70. 19919878 10.1016/j.thromres.2009.09.009

[14] Winegar DA , Sherif B , Pate V , DeMaria EJ . Venous thromboembolism after bariatric surgery performed by Bariatric Surgery Center of Excellence Participants: analysis of the Bariatric Outcomes Longitudinal Database. Surg Obes Relat Dis 2011; 7: 181–188. 21421182 10.1016/j.soard.2010.12.008

[15] Becattini C , Agnelli G , Manina G , Noya G , Rondelli F . Venous thromboembolism after laparoscopic bariatric surgery for morbid obesity: clinical burden and prevention. Surg Obes Relat Dis 2012; 8: 108–115. 22014482 10.1016/j.soard.2011.09.005

[16] Kim JW , Chun EJ , Choi SI , Park DJ , Kim HH , Bang SM *et al* A prospective study on the incidence of postoperative venous thromboembolism in Korean gastric cancer patients: an inquiry into the application of Western guidelines to Asian cancer patients. PLoS One 2013; 8: e61968. 23613988 10.1371/journal.pone.0061968 PMC3629116

[17] Song KY , Yoo HM , Kim EY , Kim JI , Yim HW , Jeon HM *et al* Optimal prophylactic method of venous thromboembolism for gastrectomy in Korean patients: an interim analysis of prospective randomized trial. Ann Surg Oncol 2014; 21: 4232–4238. 25012265 10.1245/s10434-014-3893-1

[18] Kimura Y , Oki E , Ando K , Saeki H , Kusumoto T , Maehara Y . Incidence of venous thromboembolism following laparoscopic surgery for gastrointestinal cancer: a single‐center, prospective cohort study. World J Surg 2016; 40: 309–314. 26316113 10.1007/s00268-015-3234-y

[19] Inderbitzin DT , Opitz I , Giger U , Kocher T , Krahenbuhl L. Incidence of clinical pulmonary embolism after laparoscopic surgery. Br J Surg 2007; 94: 599–603. 17330858 10.1002/bjs.5666

[20] Buchberg B , Masoomi H , Lusby K , Choi J , Barleben A , Magno C *et al* Incidence and risk factors of venous thromboembolism in colorectal surgery: does laparoscopy impart an advantage? Arch Surg 2011; 146: 739–743. 21690452 10.1001/archsurg.2011.127

[21] Shapiro R , Vogel JD , Kiran RP . Risk of postoperative venous thromboembolism after laparoscopic and open colorectal surgery: an additional benefit of the minimally invasive approach? Dis Colon Rectum 2011; 54: 1496–1502. 22067177 10.1097/DCR.0b013e31823302a1

[22] Verheijen PM , Stevenson AR , Stitz RW , Clark DA , Clark AJ , Lumley JW . Prolonged use of thromboprophylaxis may not be necessary in laparoscopic colorectal surgery. Int J Colorectal Dis 2011; 26: 755–759. 21271344 10.1007/s00384-011-1139-2

[23] Henke PK , Arya S , Pannucci C , Kubus J , Hendren S , Engelsbe M *et al* Procedure‐specific venous thromboembolism prophylaxis: a paradigm from colectomy surgery. Surgery 2012; 152: 528–536. 23021132 10.1016/j.surg.2012.07.012 PMC4496155

[24] Vedovati MC , Becattini C , Rondelli F , Boncompagni M , Camporese G , Balzarotti R *et al* A randomized study on 1‐week *versus* 4‐week prophylaxis for venous thromboembolism after laparoscopic surgery for colorectal cancer. Ann Surg 2014; 259: 665–669. 24253138 10.1097/SLA.0000000000000340

[25] Wilson MZ , Hollenbeak CS , Stewart DB . Laparoscopic colectomy is associated with a lower incidence of postoperative complications than open colectomy: a propensity score‐matched cohort analysis. Colorectal Dis 2014; 16: 382–389. 24373345 10.1111/codi.12537

[26] Haut ER , Noll K , Efron DT , Berenholz SM , Haider A , Cornwell EE III *et al* Can increased incidence of deep vein thrombosis (DVT) be used as a marker of quality of care in the absence of standardized screening? The potential effect of surveillance bias on reported DVT rates after trauma. J Trauma 2007; 63: 1132–1137. 17993962 10.1097/TA.0b013e31814856ad

[27] Loud PA , Katz DS , Bruce DA , Klippenstein DL , Grossman ZD . Deep venous thrombosis with suspected pulmonary embolism: detection with combined CT venography and pulmonary angiography. Radiology 2001; 219: 498–502. 11323478 10.1148/radiology.219.2.r01ma26498

[28] Stein PD , Fowler SE , Goodman LR , Gottschalk A , Hales CA , Hull RD *et al* Multidetector computed tomography for acute pulmonary embolism. N Engl J Med 2006; 354: 2317–2327. 16738268 10.1056/NEJMoa052367

[29] Lee LH , Gallus A , Jindal R , Wang C , Wu CC . Incidence of venous thromboembolism in Asian populations: a systematic review. Thromb Haemost 2017; 117: 2243–2260. 29212112 10.1160/TH17-02-0134

